# Inhaled bovine lactoferrin modulates the p47phox–MPO–NETosis axis in acute lung injury: implications for bioengineered nanomedicine in respiratory infections

**DOI:** 10.3389/fimmu.2026.1760949

**Published:** 2026-02-26

**Authors:** GuoAn Xiang, Di Lian, JingChao Cao, ZhongKuo Yu, YaRu Liu, XiaoXiang Hu, ShouLong Deng, Xin Li, LiXin Xie

**Affiliations:** 1 College of Pulmonary & Critical Care Medicine, The Eighth Medical Center of Chinese PLA General Hospital, Beijing, China; 2 Beijing Key Laboratory for Animal Genetic Improvement, College of Animal Science and Technology, China Agricultural University, Beijing, China; 3 National Engineering Laboratory for Animal Breeding, College of Animal Science and Technology, China Agricultural University, Beijing, China; 4 Key Laboratory of Animal Genetics and Breeding of the Ministry of Agriculture, College of Animal Science and Technology, China Agricultural University, Beijing, China; 5 College of Biological Sciences, China Agricultural University, Beijing, China; 6 National Science Center for Model Animals, China Agricultural University, Beijing, China; 7 Department of Emergency, The Third Medical Center of Chinese PLA General Hospital, Beijing, China

**Keywords:** acute lung injury, bovine lactoferrin, innate immunity, NADPH oxidase, nano-sized glycoprotein, NEtosis, respiratory infectious diseases

## Abstract

**Introduction:**

Excessive oxidative burst and dysregulated neutrophil extracellular trap (NET) formation contribute to tissue damage in acute lung injury (ALI) and are largely driven by the combined actions of NADPH oxidase 2 (NOX2) and myeloperoxidase (MPO). While lactoferrin (LTF) is a known multifunctional immunomodulatory glycoprotein, its precise role in modulating the NOX2–MPO–NETosis axis in ALI remains undefined.

**Methods:**

We employed a time-course model of lipopolysaccharide (LPS)-induced ALI in C57BL/6N mice, combined with quantitative label-free lung proteomics and downstream bioinformatic analyses to map dynamic molecular changes. At the inflammatory peak, aerosolized bovine lactoferrin (bLF) was administered in vivo, and histological lung injury, pulmonary inflammatory cytokine levels, neutrophil infiltration, and markers related to the NOX2–MPO–NETosis axis were evaluated.

**Results:**

LPS induced typical ALI pathology that peaked between days 1 and 3 (D1–D3). Proteomic and network analyses consistently highlighted NET formation as a centrally enriched early KEGG pathway and identified LTF as a key protein–protein interaction hub closely connected to p47phox (encoded by Ncf1) and MPO. Evaluation of aerosolized bLF demonstrated significant mitigation of ALI pathology, reducing lung injury, pro-inflammatory cytokines, and neutrophil recruitment. Mechanistically, bLF suppressed NETosis by reducing p47phox and MPO expression and, crucially, diminished p47phox phosphorylation in vivo, consistent with reduced NOX2 activation.

**Discussion:**

These findings identify LTF as a critical dynamic regulator of the p47phox–MPO–NETosis axis in LPS-induced ALI. They also highlight bLF as a promising candidate for further translational evaluation and support the rationale for developing bioengineered, lactoferrin-based nanomedicines aimed at modulating innate immunity and mitigating neutrophil-driven lung injury in respiratory infectious diseases.

## 1 Introduction

Acute lung injury (ALI) and its severe form, acute respiratory distress syndrome (ARDS), are fundamentally defined by diffuse alveolar damage, increased vascular permeability, and severe hypoxemia. Despite optimized ventilatory strategies, mortality rates remain stubbornly high in the absence of effective, targeted pharmacotherapy (1–3).

Neutrophils play a central role in ALI pathogenesis. They accumulate in the pulmonary microvasculature and airspaces, where they release reactive oxygen species (ROS), proteases, and neutrophil extracellular traps (NETs) (4–7). Neutrophil-mediated damage originates via the oxidative burst, centered on NADPH oxidase 2 (NOX2). Superoxide production requires NOX2 assembly from membrane and cytosolic subunits, a process critically dependent on p47phox phosphorylation (8). Myeloperoxidase (MPO), stored abundantly within azurophilic granules, subsequently converts H_2_O_2_ into hypochlorous acid (HOCl) and other potent oxidants. These MPO-derived species induce widespread cellular injury: they damage lipids, proteins, and DNA; disrupt endothelial and epithelial barriers; and drive NET formation (9, 10). Furthermore, persistent or excessive NETosis contributes to microthrombosis, impaired gas exchange, and delayed resolution of inflammation in ALI/ARDS (11). Hence, this NOX2–MPO–NETosis axis is foundational to ALI pathogenesis.

Lactoferrin (LTF) is a cationic iron-binding glycoprotein belonging to the transferrin family (12, 13). It is primarily synthesized and secreted by mucosal epithelial cells and is also both synthesized and stored within the secondary granules of neutrophils (14). This critical component of mucosal innate immunity exhibits broad antimicrobial, antiviral, anti-inflammatory, and antioxidant activity. LTF primarily functions by sequestering iron and ROS (15), yet mounting data suggest it also modulates upstream redox signaling and immune pathways (16–19). While LTF suppresses NET release in various inflammatory settings, its direct regulation of the NOX2–MPO machinery in ALI remains undetermined.

This investigation utilized a time-course, LPS-induced ALI mouse model coupled with label-free lung proteomics to map dynamic molecular changes during the onset and resolution of injury. We subsequently focused the analysis on neutrophil-related and oxidative pathways, which led to the construction of LTF-centered protein–protein interaction (PPI) networks. Finally, we evaluated aerosolized bLF *in vivo* to determine whether this exogenous agent attenuates ALI by modulating the p47phox–MPO–NETosis axis.

## 2 Materials and methods

### 2.1 Study approval

All procedures were performed in compliance with the National Guidelines for the Care and Use of Laboratory Animals (Ministry of Science and Technology, PRC) and were approved by the Committee on the Ethics of Laboratory Animals of China Agricultural University (Approval No.: AW03803202-3-1). To mitigate discomfort, both intratracheal administrations and euthanasia were executed under isoflurane anesthesia.

### 2.2 LPS-induced ALI mouse model and bLF administration

C57BL/6N mice (male and female, 8-10 weeks old) were obtained from Vital River Laboratory Animal Technology Co., Ltd. (Beijing, China). Animals were maintained under specific pathogen-free (SPF) conditions on a standardized light/dark cycle with ad libitum access to food and water. All experimental procedures were approved by the Institutional Animal Care and Use Committee of China Agricultural University (approval no. AW03803202-3-1). ALI was induced by administering lipopolysaccharide (LPS; 5 mg/kg in sterile saline; Sigma-Aldrich, USA) via a microsprayer aerosolizer (YUYAN Instrument, China). Control mice received an equal volume of saline via the same route. For the time-course model, independent cohorts of mice were exposed to LPS or saline and sacrificed at four time points after exposure: day 1, 3, 7, and 14 (D1, D3, D7, D14). At each time point, lung tissue (for histology, proteomics, qPCR, and Western blot) and/or peripheral blood cells (PBC) for flow cytometry were collected to characterize the dynamic changes in lung injury, inflammation, neutrophil recruitment, and protein expression profiles. Exogenous bLF was administered to evaluate its protective effects specifically at the early inflammatory peak (D1). bLF (cat. no. 1001102; Yulin Foodstuff Co., Ltd., Guangzhou, China), manufactured by Warrnambool Cheese and Butter Factory Company Holdings Ltd. (Warrnambool, Australia), was dissolved in sterile medium, passed through a 0.22 μm filter for sterilization (Millipore, SLGP033R), and gradually diluted in sterile medium to a final concentration of 4 mg/mL, and 50 μL of this solution (containing 200 μg bLF) was administered via pulmonary delivery. The inhaled bLF dose in this study was chosen based on prior preclinical evidence that similar lactoferrin doses are anti-inflammatory and non-toxic (20). For inhalation delivery, bLF was similarly aerosolized using a microsprayer aerosolizer. According to the manufacturer’s specifications, the device produces an aerosol with a mass median aerodynamic diameter (MMAD) of ≤30 μm, ensuring deposition in the distal conducting airways and alveolar regions of mice. Under these conditions, inhaled bLF is expected to reach the alveolar space and directly interact with resident and infiltrating immune cells at the epithelial surface. For the D1 intervention experiment, a separate set of mice was randomly as-signed to three groups (*n =* 8 per group): CON_D1 (saline control), LPS_D1 (5 mg/kg LPS), and bLF_D1 (bLF administered via intratracheal nebulization 1-2 h before LPS challenge). These animals were all sacrificed at D1, and lung tissue and bronchoalveolar or lung homogenate samples were collected for subsequent inflammation analyses.

### 2.3 Histological and immunologic analyses of lung injury and neutrophil activation

For histopathological evaluation, lung specimens were harvested and immediately fixed in 10% neutral buffered formalin, followed by paraffin embedding. Serial sections of uniform 4 μm thickness were prepared and mounted on slides, then subjected to conventional hematoxylin and eosin (H&E) staining through standardized dehydration and mounting procedures. A semi-quantitative scoring system (21) was applied to assess pulmonary injury by two independent pathologists blinded to experimental groups. The evaluation encompassed five histologic criteria: alveolar inflammatory infiltration, interstitial leukocyte accumulation, hyaline membrane formation, intra-alveolar proteinaceous debris deposition, and septal wall thickening. Each parameter was graded on an ordinal scale (0-2 points) with differential weighting based on pathological significance. The cumulative weighted scores from six randomly selected fields per section were averaged to generate a composite injury score normalized to a 0-1 range. The slides were also immune stained with anti-Ly6G (GB11229, 1:200), anti-MPO (GB15006, 1:200), to determine the accumulation and activation status of neutrophils. Images were acquired on a Nikon Eclipse CI-S microscope (Nikon, Japan) and processed with the ImageJ software (version 1.52p, National Institutes of Health, USA).

### 2.4 Single-cell suspensions processing for flow cytometry

Two types of single-cell suspensions were prepared for flow cytometric analysis. 200 μL fresh murine PBC was mixed with 1 mL ACK (Beyotime biotechnology, C3702), incubated 10 min at room temperature, centrifuged at 500 × *g* for 5 min at 4 °C, washed once with PBS and resuspended in 50 μL FACS buffer. Right lower lung lobe was minced and digested three times for 20 min at 37 °C in 1.2 mL 0.2% Collagenase I (Sigma-Aldrich, #C0130), passed through a 300 mesh filter, lysed in 1 mL ACK buffer 10 min, centrifuged at 300 × *g* for 10 min at 4 °C, washed and 5 × 10^5^ cells resuspended in 50 μL of FACS buffer.

Before staining with specific antibodies, cells were pretreated with Fc Block-2.4G2 (70-0161) to inhibit Fcγ III/II receptors. For cell surface marker analysis, cell pellets were stained with appropriate antibodies at 4 °C for 20-30 min. The following antibodies were used for staining: CD45-APCeF780 (30-F11), Ly6G-BV421 (RB6-8C5), CD16/32-Unconjugated (93). Flow cytometry was performed using a BD FACSVerse™ Cell Analyzer (BD Biosciences, USA). The acquired data were analyzed with the FlowJo software (v10).

### 2.5 Luminex liquid suspension chip detection

Luminex liquid suspension chip detection was performed by Wayen Biotechnologies (Shanghai, China). The Bio-Plex Pro Mouse Chemokine Panel 31-Plex kit was used in accordance with the manufacturer’s instructions. In brief, equal masses (45 µg) of mouse lung-lysate samples, derived from different time points and treatment groups, were incubated for 30 min in 96-well plates pre-embedded with microbeads, followed by incubation with detection antibody for 30 min. Subsequently, streptavidin-PE was added to each well for 10 min, and fluorescence signals were read using the Bio-Plex MAGPIX System (Bio-Rad).

### 2.6 Mass spectrometry analysis for label-free proteomics and bioinformatic analysis

Lungs harvested at D1, D3, D7, and D14 from NS and LPS groups in the LPS-induced ALI model were snap-frozen and stored at -80 °C until analysis. The cryopreserved tissues were subsequently subjected to data-independent acquisition (DIA) mass spectrometry at Wayen Biotechnologies (Shanghai, China) using an Orbitrap Exploris 480 interfaced with a FAIMS Pro device, following the previously established protocol. Briefly, total protein was extracted and divided into two aliquots: one for concentration determination and SDS-PAGE quality control, and the other for tryptic digestion. Following desalting, peptides were analyzed by liquid chromatography tandem mass spectrometry (LC-MS/MS). The workflow comprised two sequential stages. First, a spectral library was generated using data-dependent acquisition (DDA) on pooled peptides that had been pre-fractionated into nine discrete fractions with the Thermo Scientific High pH Reversed-Phase Peptide Fractionation Kit (Cat. No. 84868). Second, individual samples were randomized and analyzed by DIA, with parallel acquisition of MS1 and MS2 spectra. Raw DIA files were processed in Spectronaut v11.0 (Biognosys AG, 2013) for normalization and relative protein quantification. Differential abundance was assessed by t-test, with significance defined as *p* < 0.05 and fold change > 1.5; proteins meeting these criteria were defined as differentially expressed proteins (DEPs). For downstream functional analysis, DEPs were mapped to their corresponding gene symbols and used as input for Gene Ontology (GO) and Kyoto Encyclopedia of Genes and Genomes (KEGG) enrichment, as well as gene set enrichment analysis (GSEA). Nine-quadrant plot analysis, Short Time-series Expression Miner (STEM) analysis, GSEA, and heatmap visualization were performed using the OmicShare tools platform (https://www.omicshare.com/tools).

### 2.7 Western blotting

Total protein was extracted from cells using RIPA lysis buffer (Beyotime, China, P0013B) containing 1% phenylmethanesulfonyl fluoride (Beyotime, China, #ST506). Extracts containing equal quantities of protein were electrophoresed in 10% SDS–polyacrylamide gel (EpiZyme Biotechnology, # PG112), transferred onto PVDF membranes, and blocked for 1 h in Western Blocking Buffer (Beyotime, China, #P0023B) at room temperature. Antibodies used in western blotting were: LTF (Proteintech, China, #31267-1-AP), MPO (Immunoway, USA, YM8618), p47phox (Proteintech, China, #28187-1-AP), p-p47phox (Immunoway, China, #YP0828) and β-actin (QuaYad, China, #QYA10734A). Membranes were incubated with appropriate horseradish peroxi-dase-conjugated goat anti-rabbit secondary antibody (Beyotime, China, #A0208) for 2 h at room temperature. Finally, signal was detected with an integrated chemiluminescence imaging system (OI-X6Touch, Guangzhou Guangyi Biotechnology, China).

### 2.8 RNA isolation and quantitative real-time PCR

Total RNA was extracted from the lung tissue of the NS and LPS groups with HiPure Universal RNA Mini Kit (Magen, China, #R4130-03) according to the manufacturer’s instructions. Quantified mRNAs were reversely transcribed into cDNAs with the PrimeScript RT reagent Kit (Takara, China). The resulting cDNAs were quantified with SYBR Green reagent (Takara, China) by using the Applied Biosystem (ABI) 7300 PULAS (Thermo Scientific, American). The relative expression levels and quantification of mRNAs (Ltf, Mpo, Ncf1) were calculated with the 2^-△△Ct^ method. Gapdh was used as an internal control. All the primers were provided by Sangon Biotech (Shanghai, China) listed in **Table 1**.

**Table 1:** Primer sequence.

Genes	Sequence (5’→3’)
Gapdh	F: TGGCCTTCCGTGTTCCTAC
	R: GAGTTGCTGTTGAAGTCGCA
Ltf	F: GCGGGGTGGAAAATCCCTAT
	R: ACACGAGCTACACAGGTTGG
Mpo	F: CAATATGGCACGCCCAACAA
	R: TTCTCCCACCAAAACCGATCA
Ncf1	F: ACACCTTCATTCGCCATATTGC
	R: TCGGTGAATTTTCTGTAGACCAC

### 2.9 Enzyme-linked immunosorbent assays

Levels of interleukin-1β (IL-1β), interleukin-6 (IL-6), and tumor necrosis factor-α (TNF-α) in lung tissue homogenates were quantified using commercial ELISA kits (CUSABIO, China; Cat# CSB-E08054m for IL-1β, CSB-E04639m for IL-6, CSB-E04741m for TNF-α) according to the manufacturer’s instructions. Briefly, after preparing standards and samples, 100 μL of each were added to designated wells in duplicate. Following a 2 h incubation at 37 °C, wells were aspirated and sequentially incubated with 100 μL of biotin-conjugated detection antibody for 1 h and 100 μL of HRP-avidin for another 1 h at 37 °C. Between incubations, plates were washed three times with wash buffer. Color development was initiated by adding 90 μL of TMB substrate and incubating for 15-30 min at 37 °C in the dark. The reaction was terminated with 50 μL of stop solution, and optical density was immediately measured at 450 nm with reference correction at 540 nm.

### 2.10 Statistical analysis

All statistical analyses were performed using GraphPad Prism software (Version 9.5.1). All experiments included a minimum of three biological replicates per group. Data are presented as mean ± standard error of the mean (SEM). The differences within groups were analyzed using one-way analysis of variance (ANOVA). Two-tailed Student’s t-test was used for comparisons between two groups. *p* < 0.05 was considered statistically significant.

## 3 Results

### 3.1 LTF expression dynamics track neutrophil infiltration during ALI

**Figure 1:**
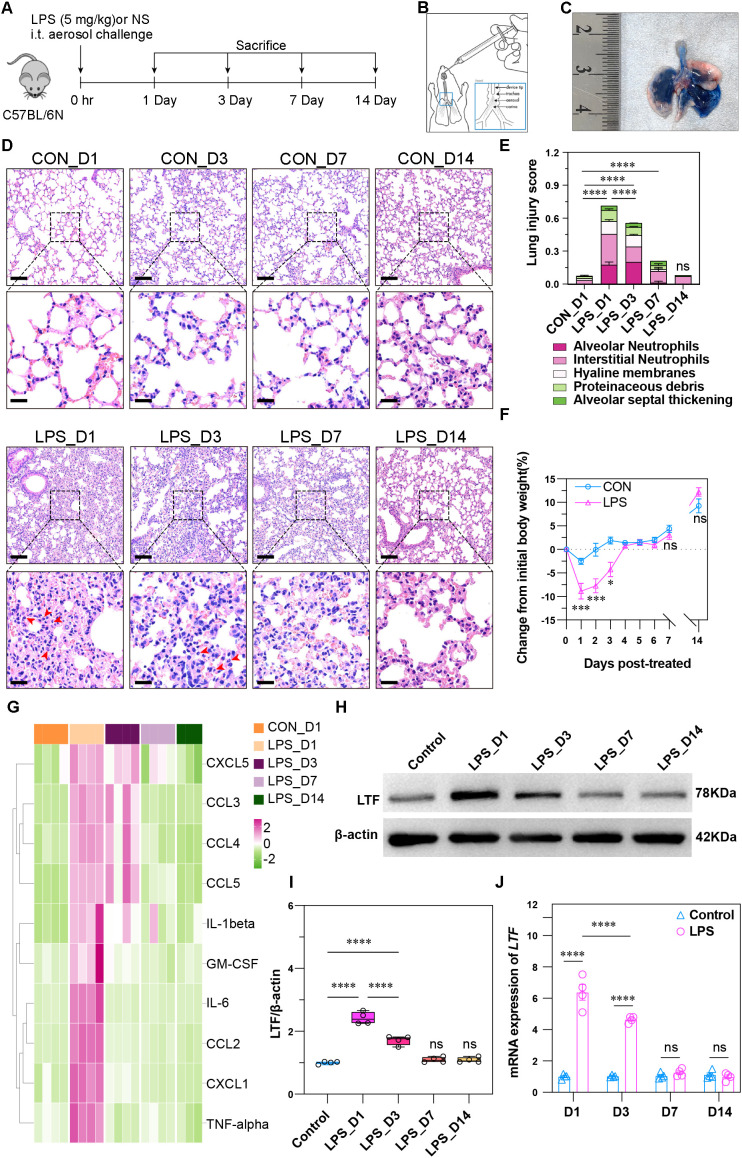
Pathological and molecular features of time-resolved LPS-induced ALI model. **(A)** Schematic of the experimental timeline for LPS-induced ALI modeling and sample collection in mice. **(B)** Illustration of pulmonary administration via a high-pressure syringe for aerosolized formulation delivery. **(C)** Trypan blue staining confirming uniform pulmonary distribution across all lung lobes. **(D)** Representative images of lung gross anatomy and H&E-stained sections at four time points after LPS stimulation. Scale bars: 100 μm (upper panel), 50 μm (lower panel). **(E)** Lung injury scores quantified by five pathological features. Peak injury at D1, sustained at D3 and D7, resolution by D14 (*n =* 4; **p < 0.01, ***p < 0.001, ****p < 0.0001, ns, not significant). **(F)** Body weight changes after LPS stimulation. Maximal loss at D1, recovery from D4 (*n =* 4; **p < 0.01, ***p < 0.001, ****p < 0.0001, ns, not significant). **(G)** Cytokine measurement using Luminex xMAP technology. Elevation of IL-1β, IL-6, TNF-α and GM-CSF at D1; CXCL5, CCL3, CCL4 and CCL5 peaked at D1 and remained high until D3. **(H-J)** LTF protein and mRNA expression peaked at D1, remained elevated at D3, normalized by D7 (*n =* 4; **p < 0.01, ***p < 0.001, ****p < 0.0001, ns: not significant).

**Figure 2:**
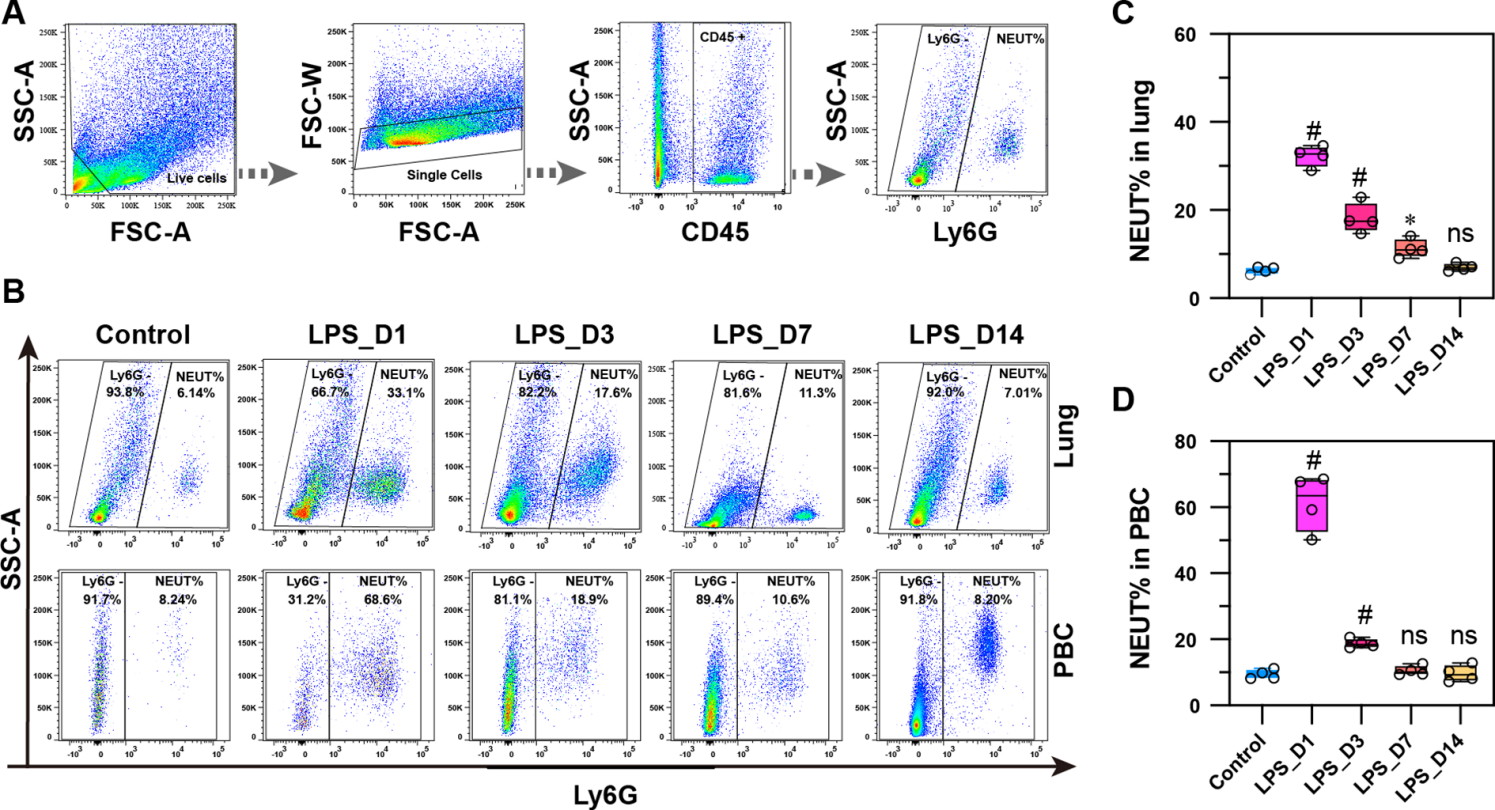
Neutrophil proportion dynamics in lung and PBC after LPS stimulation. **(A)** Gating strategy for CD45^+^Ly6G^+^ neutrophils. **(B)** Representative flow plots of lung (upper) and PBC (lower) neutrophils. **(C)** NEUT% in lung tissue peaked at D1, remained elevated through D7, resolved by D14. **(D)** NEUT% in PBC peaked at D1 and returned to baseline by D7 (*n =* 4; *p < 0.01 and #*p* < 0.001 vs. control; ns, not significant).

To delineate the pathological and molecular features of neutrophil-mediated inflammation in ALI, we utilized an ALI mouse model induced by pulmonary LPS (5 mg/kg). C57BL/6N mice received LPS or NS; subsequent analyses were conducted at D1, D3, D7, and D14 (**Figure 1A**). Pulmonary administration achieved uniform delivery across all lung lobes (**Figures 1B, C**, confirmed via Trypan blue staining). We evaluated the dynamic progression of injury by assessing lung histology based on five key features: alveolar neutrophils, interstitial neutrophils, hyaline membranes, proteinaceous airspace debris, and alveolar septal thickening. Histopathological evaluation confirmed a temporal injury progression following LPS challenge (**Figures 1D, E**). Injury peaked at D1, characterized by prominent neutrophilic infiltration (alveolar and interstitial), hyaline membrane formation, and dense proteinaceous airspace debris. While severity attenuated, significant injury, including ongoing inflammation and alveolar septal thickening, was sustained through D3 and D7. Resolution was substantial by D14, with histology returning to near control levels. Body weight dynamics following LPS stimulation mirrored this progression (**Figure 1F**), exhibiting maximal loss at D1, partial recovery by D3, and normalization relative to controls from D4 to D14. To delineate the inflammatory cascade, we employed a Luminex assay on lung homogenates (**Figure 1G**). Pro-inflammatory cytokines (IL-1β, IL-6, TNF-α, GM-CSF) were elevated at D1. In contrast, key chemokines (CXCL5, CCL3, CCL4, CCL5) peaked on D1 but persisted at elevated levels until D3. Endogenous LTF expression dynamics were also characterized post-LPS challenge. Western blot analysis demonstrated that LTF protein peaked sharply at D1, sustained high levels through D3, and normalized by D7. This trend was corroborated by a congruent mRNA profile derived from qPCR analysis (**Figures 1H–J**). Flow cytometric analysis of lung tissue and PBC assessed neutrophil infiltration. Using the CD45^+^Ly6G^+^ gating strategy (**Figure 2A**), the neutrophil proportion (NEUT%) peaked at D1 in both compartments (**Figures 2B–D**). While remaining elevated at D3, a statistically significant increase compared to controls was sustained solely in the lungs through D7, achieving complete resolution by D14. Collectively, these data establish that neutrophil chemotaxis and activation within both the pulmonary tissue and peripheral circulation constitute a central pathological process in LPS-induced ALI.

### 3.2 Integrated proteomic analysis reveals a core module of neutrophil-mediated immunity dynamically regulated during injury progression

**Figure 3:**
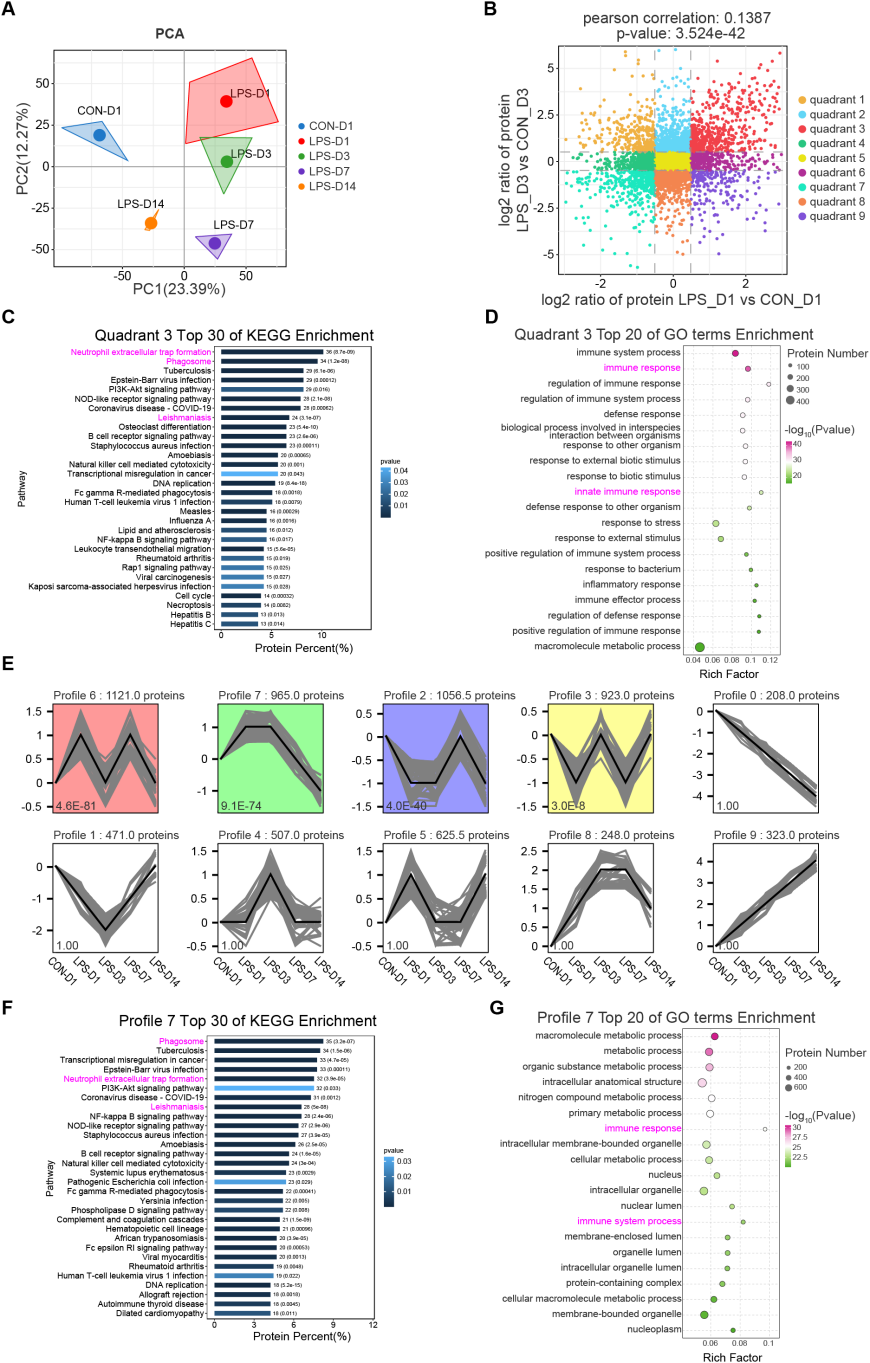
Integrated proteomic analysis reveals neutrophil-mediated pathways in LPS-induced lung injury. **(A)** PCA showing time-dependent resolution of LPS-induced injury along PC1, with greatest separation from control at D1 and gradual recovery thereafter. **(B)** Nine-quadrant analysis of LPS_D1/CON_D1 and LPS_D3/CON_D3 comparisons identifying the “quadrant 3” protein set, followed by KEGG **(C)** and GO **(D)** enrichment analyses (top 30 KEGG pathways/top 20 GO terms by p-value). **(E)** STEM time-series analysis identifying 10 expression profiles, including profile 7 (965 proteins, *p =*9.1 × 10^−74^) showing early upregulation (D1-D3) and subsequent downregulation (D7-D14). KEGG **(F)** and GO **(G)** analyses of profile 7 consistently highlighted “NET formation” and immune response pathways.

To obtain a high-resolution proteomic map of mouse lungs under normal and pathological conditions, we performed data-independent acquisition (DIA) mass spectrometry on lung tissues from WT mice collected on days 1, 3, 7, and 14 after LPS or normal saline (NS) administration. Principal component analysis (PCA) of the protein intensity data was first conducted to assess sample homogeneity and intergroup differences (**Figure 3A**). The analysis revealed a clear, time-dependent recession of the LPS-induced inflammatory response along principal component 1 (PC1). Specifically, the LPS group was most distant from the NS control at day 1, and this separation progressively diminished over time, indicating that the inflammatory response peaked initially and then gradually resolved toward homeostasis.

Upon completing the differential analysis of the proteomics data, we performed a nine-quadrant analysis by applying log_2_ transformation to the protein ratios from the comparisons of LPS_D1 vs. CON_D1 and LPS_D3 vs. CON_D3 (**Figure 3B**). This identified the “quadrant 3” protein subset, which exhibited high expression in both comparative groups. The protein identifiers in this subset were converted to gene IDs, followed by KEGG functional enrichment analysis (**Figure 3C**) and GO enrichment analysis (**Figure 3D**). The results showed the top 30 KEGG pathways and top 20 GO terms ranked by p-value. KEGG analysis highlighted pathways including “NET formation”, “Phagosome”, and “Leishmaniasis”, while GO analysis emphasized terms such as “immune response” and “innate immune response.”

In parallel, we performed Short Time-series Expression Miner (STEM) temporal trend analysis on the entire set of proteins captured by mass spectrometry, which identified 10 distinct expression clusters (**Figure 3E**). Among these, profile 7 contained 965 proteins (*p* = 9.1 × 10^-74^) and exhibited a characteristic expression pattern: up-regulated at D1 and D3 after LPS stimulation, followed by down-regulation starting at D7 and further decreasing by D14. This expression trend closely mirrors the pathological features described in Result 1. To explore the functional role of this protein set in disease progression, we conducted KEGG and GO enrichment analyses on profile 7 (**Figures 3F, G**), revealing the top 30 KEGG pathways and top 20 GO terms ranked by p-value. KEGG analysis again high-lighted pathways such as “NET formation”, “Phagosome”, and “Leishmaniasis”, though the number of enriched proteins differed from the quadrant 3 results. Similarly, GO analysis reaffirmed terms including “immune response” and “innate immune response”.

Our integrated analysis of the proteomic data delineated the core protein modules upregulated in the early stage (D1, D3) of LPS-induced injury. Functional analysis revealed that these modules were predominantly linked to neutrophil activities, including “NET formation”, and contained crucial actors like LTF and MPO in profile 7. This multifaceted proteomic evidence provides robust support for the observations as mentioned earlier in this study.

### 3.3 PPI analysis reveals LTF as a key regulator of neutrophil oxidative burst in ALI

**Figure 4:**
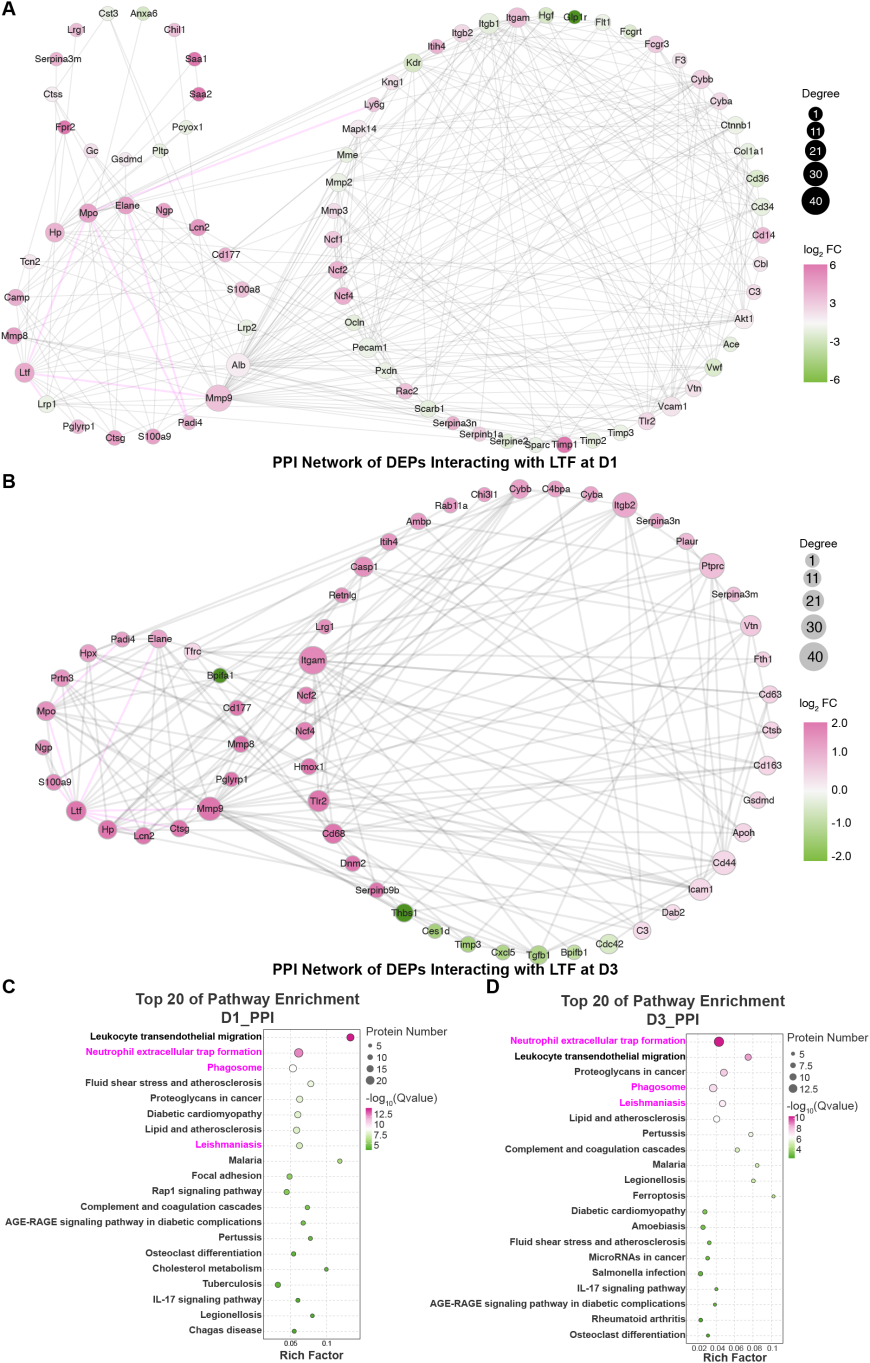
PPI network analysis reveals protein sets closely interacting with LTF at the D1 and D3 time points and identifies enriched signaling pathways. PPI analysis of differentially expressed proteins was conducted using the STRING database, and the results were visualized and analyzed in Cytoscape to construct two LTF-centered interaction networks, identifying its first- and second-order interactors at D1 **(A)** and D3 **(B)**. KEGG enrichment analysis of the protein nodes within these D1 **(C)** and D3 **(D)** networks identified the top 20 pathways by p-value.

Based on the proteomic signatures of the quadrant 3 and profile 7 protein sets, we selected the D1 and D3 time points for further investigation. The DEPs from D1 and D3 were separately and collectively subjected to PPI analysis using the STRING database, and the resulting networks were analyzed in-depth with Cytoscape. For each time point, we extracted proteins exhibiting primary and secondary interactions with LTF and constructed two LTF-centric PPI networks (**Figures 4A, B**), In both networks, we readily identified MPO along with multiple key subunits of the NADPH oxidase complex, including Ncf1 (encoding p47phox), Ncf2, and Ncf4.

**Figure 5:**
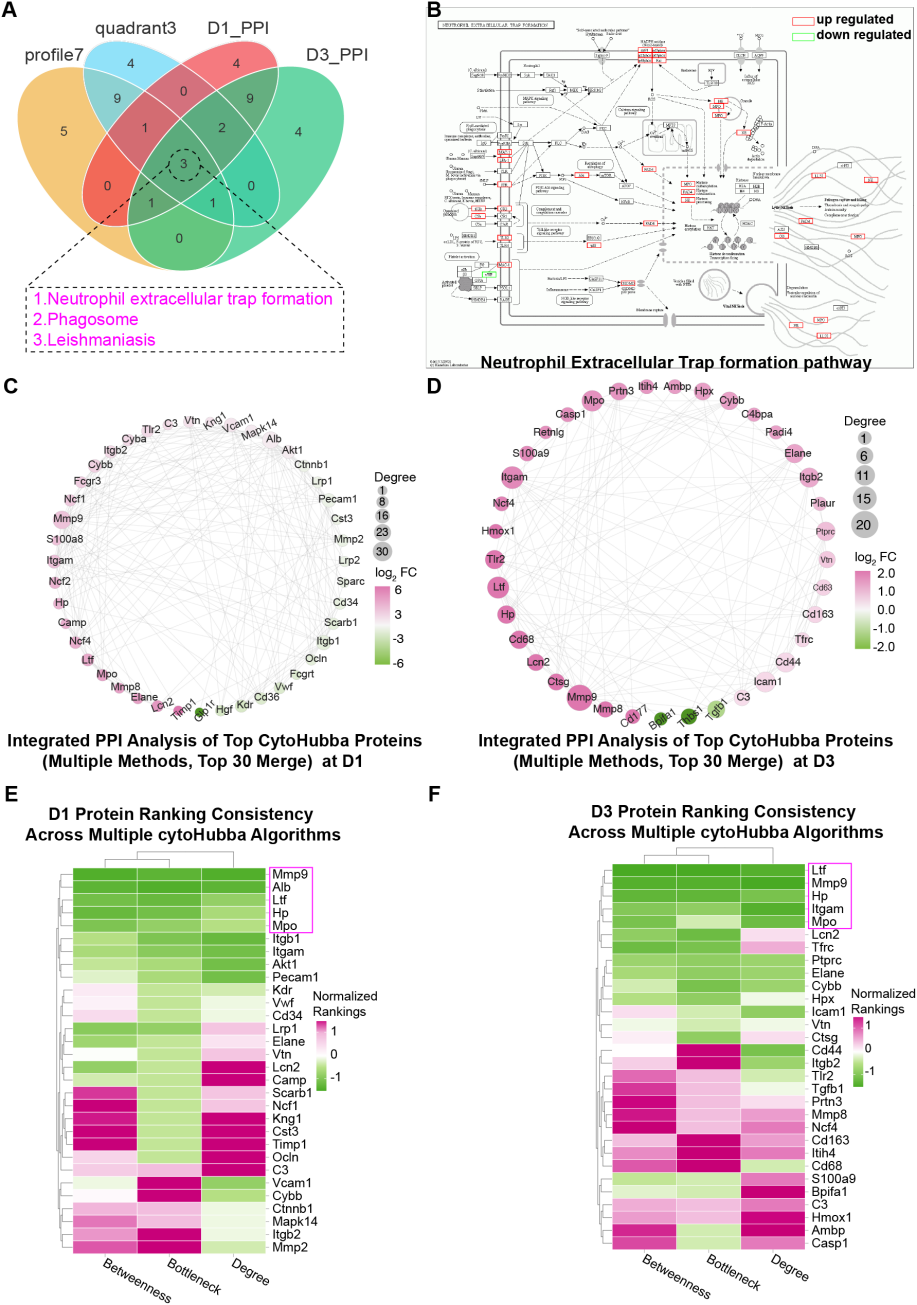
**(A)** Perform Venn diagram analysis on the pathways obtained from KEGG enrichment analysis of the protein sets screened through quadrant 3, profile 7, and the protein sets interacting with LTF at D1 and D3, respectively, yielding three pathways: “NET formation,” “Phagosome,” and “Leishmaniasis”. **(B)** The pathway map for “NET formation” at D1 is displayed, with upregulated (red box) and downregulated (green box) proteins highlighted. Protein-protein interaction (PPI) networks of the top 30 proteins identified by three algorithms in the cytoHubba plugin for the LTF-interacting protein sets at D1 **(C)** and D3 **(D)** time points. Heatmap clustering of the protein rankings from the three algorithms. The top 5 consistently highly ranked proteins (LTF, MPO, MMP9, HP, ALB) are shown for D1 **(E)** and D3 **(F)**.

The protein sets from the D1 and D3 PPI networks were then separately analyzed by KEGG enrichment, yielding the top 20 mechanistic pathways ranked by p-value (**Figures 4C, D**). A Venn diagram analysis was performed to compare these two KEGG results with those from the quadrant 3 and profile 7 protein sets (**Figure 5A**). The intersection identified only three shared pathways: “NET formation”, “Phagosome”, and “Leishmaniasis”. **Figure 5B** displays the “NET formation” pathway map, with upregulated and downregulated proteins marked in red and green, respectively. Key NET components such as MPO and ELANE, as well as multiple subunits of the upstream NADPH oxidase complex (e.g., p47phox), were observed to be upregulated.

Subsequently, we applied three algorithms via the cytoHubba plugin in Cytoscape to score the two LTF-centric PPI networks. The top 30 proteins from each scoring method were selected, and their union was taken to generate the high-ranking LTF-interacting protein networks for D1 and D3 (**Figures 5C, D**). The analysis confirmed a primary interaction between LTF and MPO, which exhibited high combined scores and edge betweenness in both networks (D1 = 0.997, D3 = 0.997). Further clustering based on these scores identified five core proteins (MMP9, ALB, LTF, HP, MPO) that consistently ranked within the top five across all three algorithms (**Figures 5E, F**).

These results lead us to hypothesize that during the pathogenesis of LPS-induced lung injury, LTF directly interacts with MPO and may indirectly regulate the downstream oxidative cascade of the NADPH oxidase system, thereby influencing NETosis.

### 3.4 GSEA validates NET formation as a core pathway in early ALI

To further validate the protein expression and functional enrichment in lung tissues during LPS-induced lung injury, we performed GSEA to identify differentially expressed proteins between normal and diseased states. The analysis utilized all detected proteins without prefiltering, with the protein ranking based on the Signal2Noise method. Default parameters were maintained throughout the analysis, which was conducted using the OmicShare platform (https://www.omicshare.com/tools).

**Figure 6:**
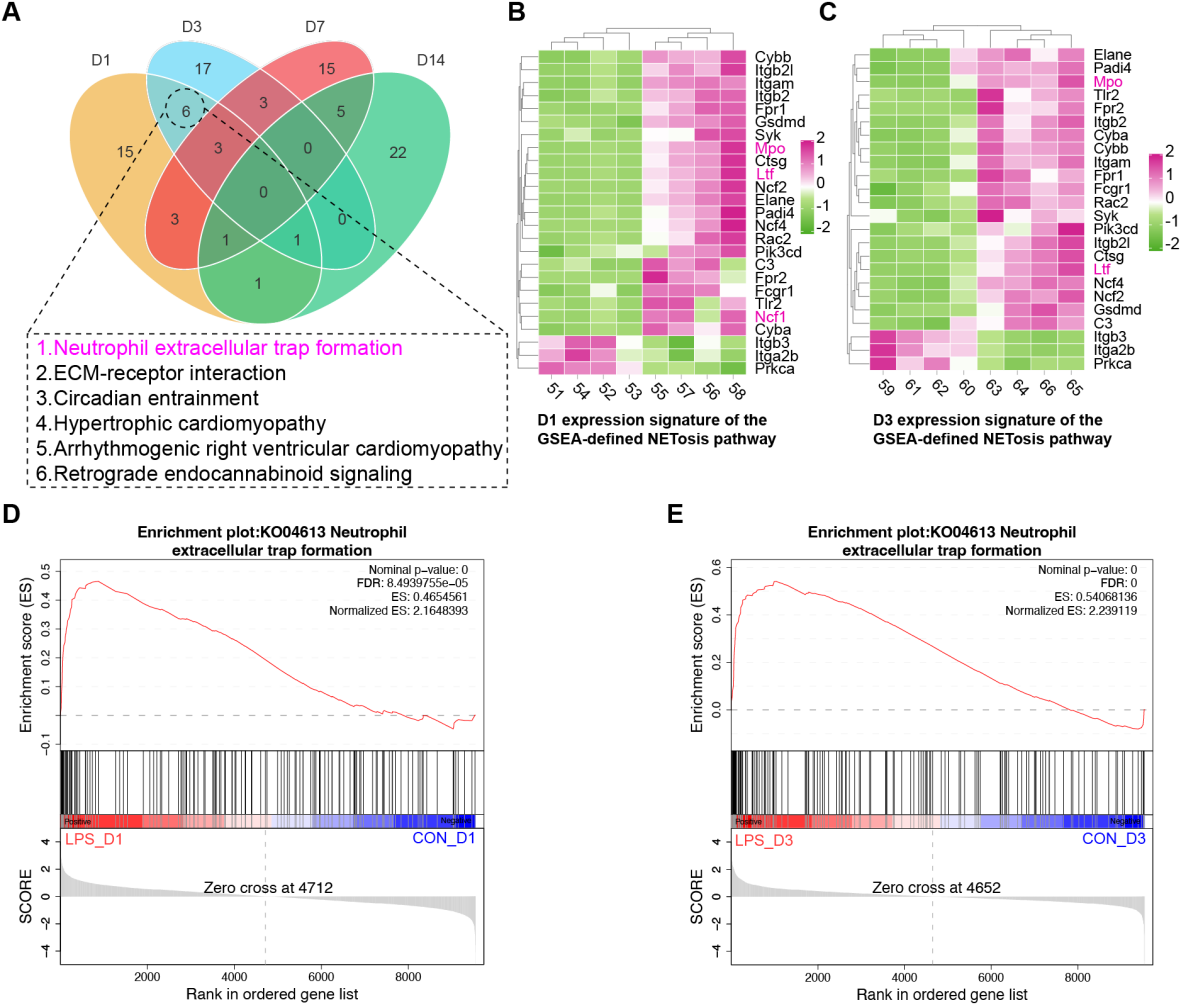
GSEA was performed on the entire list of captured proteins from all four time points, regardless of their differential expression status, using the KEGG pathway database. A Venn diagram analysis of all enriched pathways across the time points **(A)** identified five pathways uniquely and commonly enriched at both D1 and D3, including “NET formation”, “ECM-receptor interaction”, “Circadian entrainment”, “Hypertrophic cardiomyopathy”, “Arrhythmogenic right ventricular cardiomyopathy”, and “Retrograde endocannabinoid signaling”. Notably, “NET formation” was the only pathway that overlapped with those identified in previous analyses. The proteins enriched in this pathway and LTF are displayed in a heatmap **(B, C)**, showing the upregulation of proteins such as MPO, p47phox, PADI4, NCF2, and NCF4. The GSEA enrichment score plots for the “NET formation” pathway at the D1 and D3 time points are shown in **(D, E)**, respectively.

GSEA of KEGG pathways across four time points revealed six pathways exclusively enriched at both D1 and D3 but not at other time points: NET formation, ECM-receptor interaction, Circadian entrainment, Hypertrophic cardiomyopathy, Arrhythmogenic right ventricular cardiomyopathy, and Retrograde endocannabinoid signaling. Venn diagram analysis (**Figure 6A**) demonstrated that NET formation was the only pathway overlapping with our previously identified mechanisms. A heatmap visualizing proteins enriched in this pathway, along with Ltf, showed upregulated expression of Mpo, Elane, Padi4, Ncf1, Ncf2, and Ncf4 at D1, with sustained elevation at D3 (**Figures 6B, C**). The enrichment score plot (**Figures 6D, E**) further confirmed that LPS robustly activated the NET formation pathway at both D1 and D3.

These results further support our hypothesis that neutrophils play a crucial role in the pathological progression at D1 and D3 following LPS challenge. LTF may emerge as a pivotal regulator in this process, directly interacting with MPO and influencing the oxidative stress circuitry through modulation of p47phox, ultimately affecting the activation of NET formation. The LTF-p47phox-MPO axis appears to function as a switchable mechanism that can transition between pro-inflammatory injury and anti-inflammatory repair—a dynamic behavior reflecting the dual nature of oxidative stress. During the early inflammatory phase (D1), excessive antimicrobial and proinflammatory responses may cause tissue damage, while the reactive upregulation of LTF likely establishes a foundation for the subsequent shift toward repair in later stages.

### 3.5 Functional validation of the LTF-p47phox-MPO axis at the inflammatory peak (D1)

Since our previous results consistently identified D1 as the peak inflammatory time point in LPS-induced lung injury in this experimental model, we selected this time point for functional validation of the LTF-p47phox-MPO axis. We again utilized C57BL/6N mice, establishing three experimental groups: CON_D1 (normal saline control), LPS_D1 (5 mg/kg LPS), and bLF_D1 (pre-administered nebulized exogenous bovine lactoferrin at a dose of 200 μg in a volume of 50 μL, 1-2 h prior to LPS challenge). Tissues were collected at D1 for subsequent analyses.

**Figure 7:**
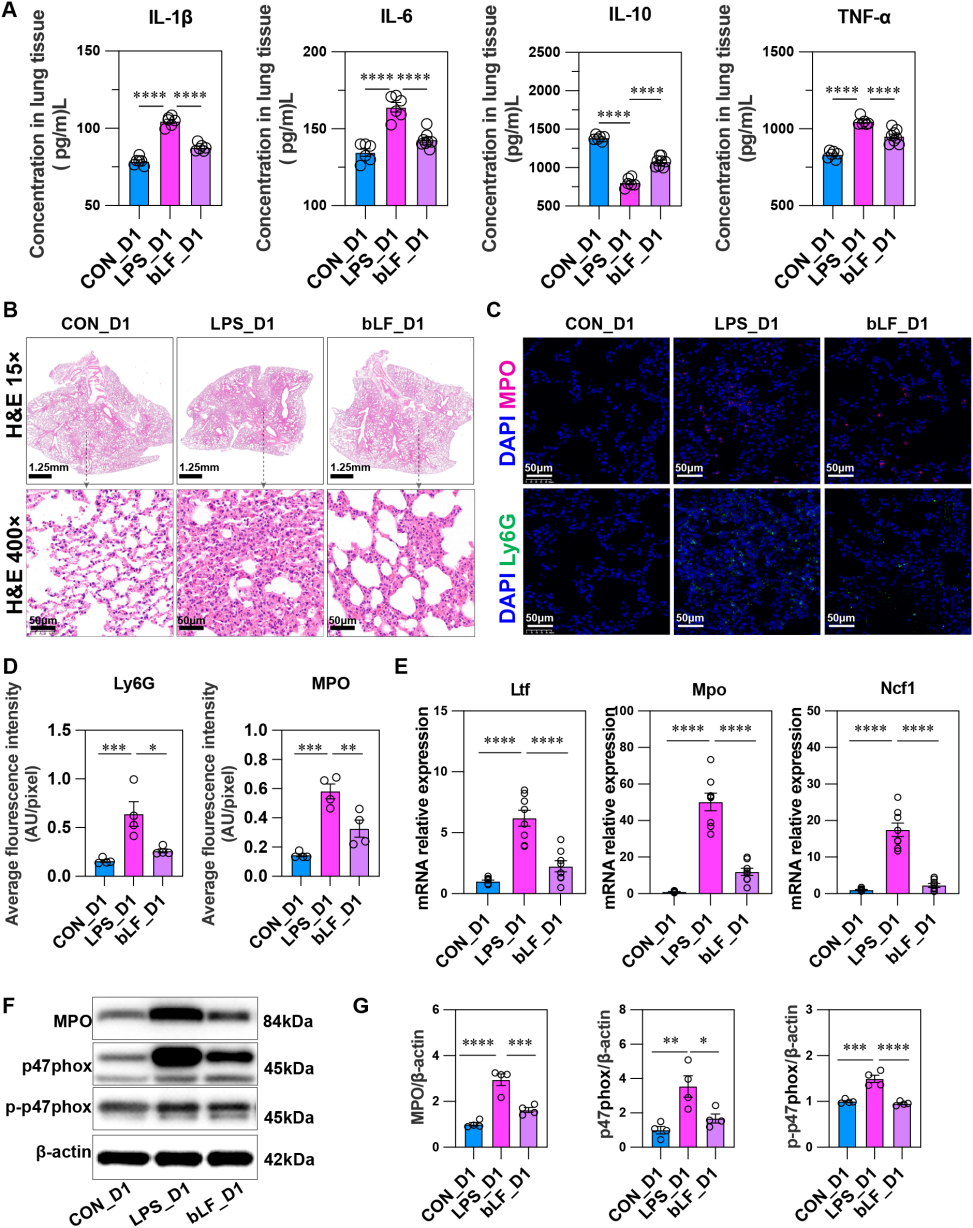
Exogenous bLF attenuates LPS-induced acute lung injury and oxidative stress response at D1. Mice were divided into three groups (*n =* 8 per group): CON_D1 (saline control), LPS_D1 (5 mg/kg LPS), and bLF_D1 (bLF administered via pulmonary delivery 1-2 h before LPS). **(A)** ELISA of lung homogenates (*n =* 8) showing that LPS-induced elevations in IL-1β, IL-6, and TNF-α were reduced by bLF treatment, while the decreased anti-inflammatory cytokine IL-10 was restored and increased after bLF administration. **(B)** H&E staining (*n =* 8) demonstrating that alveolar hemorrhage, neutrophil infiltration, and interstitial thickening in LPS_D1 lungs were alleviated in the bLF_D1 group. **(C, D)** Immunofluorescence staining (*n =* 8) of Ly6G and MPO revealed increased fluorescence intensity following LPS stimulation, which was suppressed by bLF treatment. **(E, F)** qPCR analysis of lung tissue (*n =* 8) indicating that LPS upregulates the mRNA expression of Ltf, Mpo, Ncf1, while bLF treatment reversed these changes. **(G, H)** Western blot analysis (*n =* 4) confirmed that protein levels of MPO, p47phox, and p-p47phox were elevated in the LPS_D1 group and downregulated after bLF treatment. *p < 0.01, ***p < 0.001, ****p < 0.0001, ns, not significant.

ELISA of lung homogenates (**Figure 7A**) showed that IL-1β, IL-6, and TNF-α levels were elevated after LPS stimulation, and this increase was attenuated by bLF treatment, indicating that exogenous bLF reduced the LPS-induced inflammatory response.

Histopathological analysis (**Figures 7B–D**) revealed that alveolar hemorrhage, neutrophil infiltration, and interstitial thickening observed in the LPS group were ameliorated in the bLF_D1 group. Immunofluorescence staining further demonstrated that the fluorescence intensity of Ly6G and MPO^+^ – markers representative of neutrophils – was markedly increased after LPS but reduced by bLF treatment, confirming that exogenous bLF decreased LPS-induced neutrophil infiltration.

Concurrently, mRNA quantification of several genes in lung tissue (**Figure 7E**) indicated that Ltf, Mpo, Ncf1 were upregulated at D1 after LPS stimulation, and exogenous bLF supplementation mitigated these changes. Western blot results (**Figures 7F, G**) similarly demonstrated that MPO, p47phox, and phosphorylated p47phox (p-p47phox) protein levels increased in LPS-induced lung injury, and bLF significantly downregulated their expression.

These molecular and biological data from the animal experiments robustly confirm our hypothesis: neutrophils play a critical role in the early pathological process following LPS challenge. LTF, a protective gene, is reactively upregulated during inflammatory injury and serves a vital function, likely by directly interacting with MPO and influencing the oxidative stress circuit via modulation of p47phox, ultimately regulating the activation of NETosis. The p47phox–MPO–NETosis axis acts as a switchable mechanism capable of transitioning between pro-inflammatory injury and anti-inflammatory repair – a dynamic behavior reflecting the dual nature of oxidative stress. In the early inflammatory phase (D1), excessive antimicrobial and pro-inflammatory responses may cause tissue damage. Notably, exogenous bLF supplementation exerts biological effects similar to endogenous LTF, not only downregulating the initiator of the oxidative stress cascade, p47phox, in LPS-induced lung injury, but also reducing the downstream effector, MPO, ultimately mitigating the severity of injury and playing a key role in promoting repair.

## 4 Discussion

This study utilized a dynamic LPS-induced ALI model coupled with quantitative proteomics and functional *in vivo* intervention to dissect lactoferrin’s role in the pathogenic axis defined by neutrophil-mediated oxidative stress and NETosis during early lung injury. Our key findings indicate that LPS triggers an early inflammatory surge, characterized by pronounced neutrophil recruitment and a transient upregulation of endogenous LTF expression at D1 and D3. Furthermore, multilayer bioinformatic analysis consistently identified LTF as a hub protein central to neutrophil activation and NET formation pathways, as reflected by network connectivity to p47phox and MPO. Notably, NET formation emerged as a KEGG pathway that repeatedly surfaced at the intersection of our proteomic analyses and showed robust enrichment specifically at the D1 and D3 time points. Functional validation in the LPS-induced ALI mouse model at D1 demonstrated that aerosolized bLF markedly attenuated lung injury, dampened pulmonary inflammation, reduced neutrophil infiltration, downregulated endogenous Ltf expression, suppressed Ncf1 and Mpo transcript levels, and reduced p47phox (both total and phosphorylated forms) and MPO protein levels. Collectively, these data substantiate the role of LTF as a dynamic regulator of the p47phox–MPO–NETosis axis during ALI.

The study critically employed a time-course model, departing from standard single terminal time points. Histology, cytokine profiling, and flow cytometry demonstrated rapid LPS-induced injury and inflammation, peaking at D1-D3 and resolving by D7-D14. Proteomic analysis mirrored this trajectory: PCA separated NS and LPS groups along the D1→D3→D7→D14 sequence, while STEM clustering identified proteins exhibiting initial upregulation followed by decline. NET formation was highly enriched among D1 and D3 DEPs, specifically within this early upregulated cluster, indicating tight coupling to the acute phase of injury rather than late repair mechanisms. These findings underscore the necessity of timely ALI/ARDS intervention, as the targetable pathogenic cascade is maximally active within the initial 24-72 h post-insult—precisely the window modeled.

Neutrophil recruitment and endogenous LTF induction exhibited tight temporal alignment. Flow cytometry revealed a rapid influx of CD45^+^Ly6G^+^ neutrophils into both lung tissue and PBC at D1 and D3, returning to baseline by D7. Concomitantly, Ltf mRNA and LTF protein markedly increased at D1 and D3, subsequently declining. This kinetics suggests LTF functions as an early component of the pulmonary response to profound neutrophilic inflammation and oxidative stress. Previous studies established that LTF is stored in neutrophil secondary granules (released upon degranulation) and can be expressed *de novo* under inflammatory conditions (14, 22, 23). Our data extend this knowledge by demonstrating that pulmonary LTF expression precisely parallels neutrophil influx in ALI and constitutes a key element within a broader neutrophil-related proteomic signature.

It is increasingly recognized that the relationship between LTF and neutrophils is complex and reciprocal, involving mechanisms that are not limited to antioxidant protection. Neutrophil activation leads to the release of LTF, MPO, and elastase from granules, contributing directly to the antimicrobial and immunomodulatory activities within the local tissue microenvironment (12, 14, 15). Conversely, LTF exerts a regulatory feedback loop on neutrophils, modulating their activation, adhesion, and effector functions. Reports indicate LTF limits neutrophil chemotaxis and endothelial adhesion, curtails degranulation and oxidative burst, and inhibits NET release across various models (24, 25). Mechanistically, LTF interacts with specific myeloid cell receptors (e.g., LRP1), initiating downstream signaling that promotes a less damaging neutrophil phenotype (13). This feedback regulatory role aligns with our finding that aerosolized bLF reduces pulmonary neutrophil recruitment and lowers NETosis-associated markers.

Within this context, MPO emerges as a pivotal effector at the intersection of neutrophil activation, oxidative stress, and NETosis. MPO is a highly cationic heme-containing peroxidase, constituting approximately 5% of neutrophil dry weight, stored primarily in azurophilic granules, and expressed marginally in monocytes (26–28). Its heme prosthetic group confers potent haloperoxidase activity, enabling MPO to utilize H_2_O_2_—generated by NOX2 under physiological conditions—to convert chloride, thiocyanate, and other halides into strong oxidants such as HOCl, HOBr, and HOSCN (29–31). These oxidants are crucial for killing ingested pathogens within the phagosome and are also incorporated into NETs, where they stabilize NET structures and drive NETosis (32, 33). However, their massive accumulation at inflammatory sites delays regenerative healing and initiates pathologic inflammation. The uncontrolled oxidative burst, driven primarily by NOX2, is a critical upstream mechanism of tissue damage (34, 35). High HOCl concentrations deplete nitric oxide (NO) and provoke endothelial dysfunction; meanwhile, indiscriminate oxidation of proteins, lipids, and nucleic acids activates inflammatory signaling cascades like NF-κB and NLRP3. This amplification of oxidative stress propels the progression of chronic inflammatory diseases, including cardiovascular, neurodegenerative, and acute kidney diseases (26). Although MPO possesses diverse biological actions, its best-characterized role remains its function as a downstream effector of NOX2 (36, 37). The H_2_O_2_ generated by NOX2 serves as the obligatory substrate for MPO to produce HOCl, establishing a critical functional link between these two oxidative systems both inside and outside the cell. For instance, in experimental Parkinson’s disease, mitochondrial over-induction of NOX4 in hippocampal astrocytes triggers an H_2_O_2_ burst that upregulates MPO and osteopontin (OPN), thereby aggravating mitochondrial dysfunction and neuroinflammation (38). Pharmacological NOX2 inhibitors effectively interrupt this axis, attenuating lipid peroxidation and curbing inflammatory infiltration, which consequently suppresses MPO-related oxidative injury (39). Consequently, targeting the NOX2–MPO axis emerges as a promising therapeutic strategy for a broad spectrum of inflammatory disorders.

Our proteomic and network analyses identify LTF as a central component linking neutrophil activation and oxidative injury within the NOX2–MPO–NETosis framework. Proteomic PPI networks revealed that LTF is directly or closely associated with key components of neutrophil oxidative burst and NETosis, including p47phox and MPO. Consistent with this, other canonical NET-related proteins within the same module, such as PADI4 and neutrophil ELANE, also showed marked upregulation at D1-D3, together with coordinated changes in several actin cytoskeleton–regulating components annotated in the NET formation pathway. Additionally, the KEGG pathway of NET formation was repeatedly and robustly enriched across both globally differential protein sets and LTF-centered subnetworks, with this enrichment being time-resolved at D1 and D3. Collectively, these multilayered findings reinforce the notion that LTF is intricately embedded within—and may functionally restrain—the NOX2–MPO–NETosis cascade with which it interacts. Furthermore, the aerosol inhalation route used in this study is mechanistically relevant. Local delivery enables high bLF concentrations in the airways and alveolar space while minimizing systemic exposure, thereby allowing direct engagement with resident and infiltrating neutrophils in the pulmonary microenvironment. Coupled with LTF’s favorable safety profile as an endogenous glycoprotein, these features provide a strong rationale for considering inhaled bLF as a preventive or early therapeutic adjunct in high-risk ALI settings, particularly during the early 24–72 h window when neutrophil-driven oxidative stress and NETosis are at their peak.

An important question that merits further discussion is by which primary mechanism bLF exerts its inhibitory effects on p47phox phosphorylation and MPO activity. Previous studies indicate that LTF can serve a dual function as both a targeting ligand and an iron chelating component when incorporated into specific nanoparticles along with other antioxidants. This strategy harnesses the synergistic antioxidant activity between lactoferrin and the co-loaded agents to effectively scavenge ROS, thereby offering a combined therapeutic approach for the treatment of dry age-related macular degeneration (40). Multiple *in vivo* and *in vitro* studies have shown that LTF can suppress the TLR–NF-κB/MAPK inflammatory axis (41, 42) and activate the Keap1–Nrf2 antioxidant axis (43, 44), leading to an overall reduction in pro-inflammatory cytokine production and intracellular ROS levels, and thus attenuating oxidative stress upstream at the microenvironmental level. At the same time, NF-κB and p38/JNK signaling pathways themselves are recognized as important upstream regulators of the NADPH oxidase complex (45, 46), and activation of Nrf2 is considered to negatively regulate the expression of NOX2-related proteins (47), thereby restraining persistent oxidative bursts. MPO, as a downstream effector enzyme that utilizes H_2_O_2_ generated by NOX2, has its expression and release similarly regulated by the degree of neutrophil activation and the local inflammatory signaling milieu. Transcription factors such as NF-κB and STAT can modulate Mpo transcription, driving upregulation of MPO expression in neutrophils under highly inflammatory conditions (48). MPO release depends on neutrophil degranulation and NETosis (37), processes that themselves represent the downstream outcome of TLR/NOD signaling cascades and NOX2-driven oxidative bursts (49). In the present study, we observed that aerosolized bLF markedly reduced Ncf1 mRNA levels, a decrease in total p47phox protein, and a marked reduction in its phosphorylation level, accompanied by concomitant decreases in Mpo transcript and MPO protein expression. Taken together, this pattern of changes is more consistent with the notion that bLF primarily acts by modulating upstream signaling events that govern NOX2 complex assembly and activation, thereby indirectly suppressing p47phox phosphorylation, reducing NOX2-dependent H_2_O_2_ generation, and attenuating neutrophil degranulation and MPO release, ultimately limiting NET formation. Compared with classical NETosis inhibitors such as DNase I, which primarily clear extracellular DNA, or PAD4 inhibitors that block histone citrullination at the execution phase of NETosis, bLF appears to intervene further upstream by dampening p47phox and MPO induction. This upstream positioning provides a mechanistic rationale for considering bLF as a complementary strategy to existing NET-targeted approaches, rather than a simple functional mimic.

Beyond its direct immunomodulatory role, LTF holds considerable promise as a platform for bioengineered nanomedicine. Its ability to specifically bind cell surface receptors allows it to function not only as a therapeutic agent but also as a versatile, low-immunogenicity targeting ligand or biodegradable nanocarrier (50). LTF can be integrated into various nanostructures, such as liposomes, poly(lactic-co-glycolic acid) (PLGA), and metal-organic frameworks (MOFs), to create platforms that combine active targeting with synergistic pharmacology (50–53). Such strategies would constitute a transformative advance in nano-immunotherapy for inflammatory and other complex diseases.

Several limitations should be noted. First, all experiments were performed in a single LPS-induced ALI model, which, although neutrophil-dominant, does not fully mirror the etiologic and pathophysiologic heterogeneity of human ARDS. Second, our conclusions regarding NETosis are based on pathway enrichment and changes in MPO/Ly6G and NOX2-related molecules, without direct measurement of other NET-specific markers such as cell-free DNA, nucleosomes, citrullinated histones, or MPO–DNA colocalization, so the findings should be interpreted as indirect, pathway-level evidence of attenuated NETosis rather than definitive structural proof.

## 5 Conclusions

Using a time-resolved LPS-induced ALI model, this study shows that neutrophil recruitment, NOX2-dependent oxidative stress, and NET formation dominate the early phase of lung injury, thereby establishing the pathogenic milieu that LTF modulates. Aerosolized bLF markedly attenuated lung injury and pulmonary inflammation, reduced neutrophil infiltration, and downregulated endogenous Ltf, Ncf1 and Mpo transcripts, accompanied by decreased p47phox phosphorylation and lower MPO protein levels. These findings support a model in which LTF functions as a dynamic upstream regulator of the p47phox–MPO–NETosis axis and related inflammatory signaling, thereby limiting neutrophil-driven oxidative stress damage in ALI. Moreover, as a naturally occurring, nano-sized glycoprotein with a favorable safety record and feasible lung-delivery profile, bLF emerges as a mechanistically supported candidate for further translational evaluation, including exploration as a nanoengineerable scaffold for targeted modulation of innate immunity in respiratory inflammatory and infectious diseases.

## Data Availability

The data presented in the study are deposited in the ProteomeXchange Consortium via the iProX partner repository(54, 55), accession number PXD071688.
